# Enhancement of Risk for Lyme Disease by Landscape Connectivity, New York, New York, USA

**DOI:** 10.3201/eid2506.181741

**Published:** 2019-06

**Authors:** Meredith C. VanAcker, Eliza A.H. Little, Goudarz Molaei, Waheed I. Bajwa, Maria A. Diuk-Wasser

**Affiliations:** Columbia University, New York, New York, USA (M.C. VanAcker, M.A. Diuk-Wasser);; Connecticut Agricultural Experiment Station, New Haven, Connecticut, USA (E.A.H. Little, G. Molaei);; Yale University, New Haven (G. Molaei);; New York City Department of Health and Mental Hygiene, New York, New York, USA (W.I. Bajwa)

**Keywords:** Ixodes scapularis, ticks, Borrelia burgdorferi, bacteria, enhancement, fragmentation, emergence, landscape connectivity, Lyme disease, urban Lyme disease, disease risk, vector-borne infections, tick-borne infections, New York City, New York, Staten Island, United States

## Abstract

Most tickborne disease studies in the United States are conducted in low-intensity residential development and forested areas, leaving much unknown about urban infection risks. To understand Lyme disease risk in New York, New York, USA, we conducted tick surveys in 24 parks throughout all 5 boroughs and assessed how park connectivity and landscape composition contribute to *Ixodes scapularis* tick nymphal densities and *Borrelia burgdorferi* infection. We used circuit theory models to determine how parks differentially maintain landscape connectivity for white-tailed deer, the reproductive host for *I. scapularis* ticks. We found forested parks with vegetated buffers and increased connectivity had higher nymph densities, and the degree of park connectivity strongly determined *B. burgdorferi* nymphal infection prevalence. Our study challenges the perspective that tickborne disease risk is restricted to suburban and natural settings and emphasizes the need to understand how green space design affects vector and host communities in areas of emerging urban tickborne disease.

Lyme disease, or Lyme borreliosis, is the most commonly reported arthropodborne disease in the United States and Europe (*1*). In the eastern United States, this disease is caused by *Borrelia burgdorferi* sensu stricto (hereafter *B. burgdorferi*), a spirochete transmitted by the blacklegged tick, *Ixodes scapularis,* and maintained in a horizontal transmission cycle between larval and nymphal *I. scapularis* ticks and a vertebrate reservoir host community (*2*). *I. scapularis* ticks vector 6 other tickborne pathogens, including *Babesia microti* (the cause of human babesiosis) and *Anaplasma phagocytophilum* (the cause of human granulocytic anaplasmosis). The geographic expansion of these pathogens has followed the spread of their shared vector across the northeastern and midwestern United States over the past 50 years (*3*). The range expansion of *I. scapularis* ticks is attributed to reforestation (*4*), the increase in deer populations (*4*), and climate-facilitated expansion (*5*). Although historically associated with the incursion of suburban and exurban development into rural areas (*4*), tickborne diseases are an emerging urban threat, indicated by an unprecedented increase in locally acquired cases in New York City (NYC), NY, USA (*6*), and *B. burgdorferi*–infected *I. scapularis* in Chicago, IL, USA (*7*). In contrast with several European studies on urban Lyme borreliosis (*8*), the risk for acquiring *B. burgdorferi* infection in US cities is unknown.

As tickborne diseases spread into urban areas, key issues are what ecologic and sociobehavioral conditions enable establishment of the enzootic cycle and pathogen spillover to humans. Landscape modification, such as forest fragmentation (breaking up of large continuous forests into smaller patches), has been linked to increased transmission risk for Lyme disease (*9*,*10*). Forest fragmentation increases edge habitat and might reduce host biodiversity by increasing densities of white-footed mice (*Peromyscus leucopus*), a major host for immature *I. scapularis* ticks and *B. burgdorferi*, relative to less competent hosts. Fragmentation might also favor white-tailed deer (*Odocoileus virginianus*) (hereafter deer), the reproductive host for adult *I. scapularis* ticks, through increased forage quality and predator release (*9*,*10*), and might bring humans in closer contact with forests and tick vectors, increasing human–tick contact rates (*10*). However, extreme fragmentation of suitable habitat patches within an impermeable urban matrix might decrease disease risk if connectivity is reduced to the point of limiting host and tick movement (*11*). This connectivity might be partially restored by establishing green spaces and habitat corridors within cities, which can lead to an introduction of tick populations and pathogens into new areas (*7*,*12*,*13*).

With high human densities in cities, emerging tickborne infections can cause a major public health burden (*12*). Human risk for acquiring Lyme disease is dependent on the density and infection prevalence of nymphal *I. scapularis* ticks, the hazard, or potential source of harm (*14*). Thus, understanding the drivers of vector and pathogen distribution is critical for designing effective intervention strategies. Because of increasing incidence of locally acquired Lyme disease cases on Staten Island (*6*), a borough of NYC, and the potential for expansion to other boroughs, we sought to determine the hazard posed by *I. scapularis* ticks in public parks in NYC, and characterize the effect of landscape composition and connectivity in and around parks on nymphal *I. scapularis* tick densities and *B. burgdorferi* infection prevalence.

## Materials and Methods

### Site Selection

**Figure 1:**
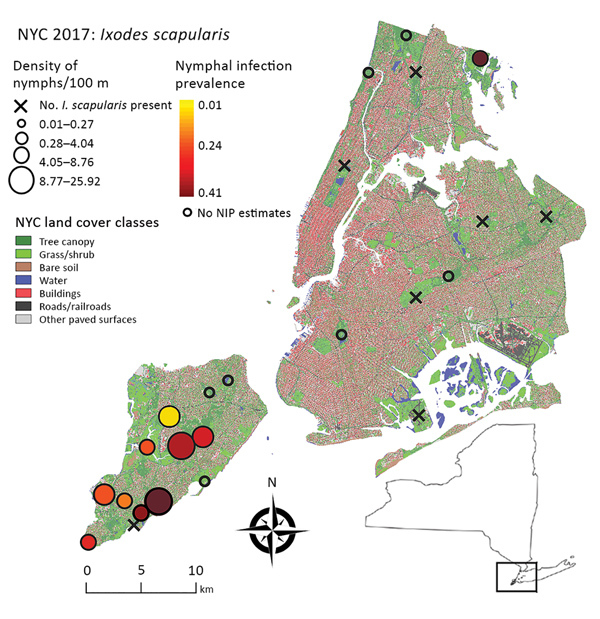
Study area for analysis of *Ixodes scapularis* nymphal tick densities and *Borrelia burgdorferi* infection prevalence, New York, New York, USA, 2017. Open circles indicate parks where tick sample size was too low to estimate nymphal infection prevalence. Inset shows location of study area in New York state. NIP, nymphal infection prevalence; NYC, New York City.

We surveyed 24 public parks in NYC (Figure 1; Table 1): 13 on Staten Island, 2 in Manhattan, 2 in Brooklyn, 3 in the Bronx, and 4 in Queens. Fifteen of these parks are included in ongoing tick surveillance by the NYC Department of Health and Mental Hygiene. The inclusion criteria we used to select the parks were location (representing all 5 boroughs) and size (26 ha–794 ha) and forest area (7 ha–433 ha) gradients.

**Table 1:** Sampling effort for study of enhancement of Lyme disease risk by landscape connectivity, New York, New York, USA*

Park	Borough	Geographic coordinates, °N, °W	No. *Ixodes scapularis* ticks tested for *Borrelia burgdorferi*/no. collected	No. sampling efforts
Alley Pond Park	Queens	40.7476, −73.7425	0/0	2
Bloomingdale Park	Staten Island	40.5334, −74.2105	39/39	2
Blue Heron Park	Staten Island	40.5314, −74.1746	54/422	2
Bronx Park	Bronx	40.8716, −73.8740	0/0	2
Central Park	Manhattan	40.7982, −73.9561	0/0	3
Clay Pit Ponds State Park Preserve	Staten Island	40.5393, −74.2321	52/156	2
Clove Lakes Park	Staten Island	40.6185, −74.1139	0/1	2
Conference House Park	Staten Island	40.5010, −74.2516	51/83	2
Floyd Bennett Field	Brooklyn	40.5983, −73.8967	0/0	2
Forest Park	Queens	40.7033, −73.8508	0/1	2
Freshkills Park	Staten Island	40.5763, −74.1835	57/82	2
Great Kills Park	Staten Island	40.5463, −74.1252	0/5	2
High Rock Park	Staten Island	40.5825, −74.1232	51/122	2
Highland Park	Queens	40.6873, −73.8871	0/0	2
Inwood Hill Park	Manhattan	40.8732, −73.9250	0/1	2
Kissena Park	Queens	40.7435, −73.8057	0/0	2
Latourette Park	Staten Island	40.5880, −74.1395	105/622	3
Lemon Creek Park	Staten Island	40.5115, −74.1977	0/0	2
Pelham Bay Park	Bronx	40.8673, −73.8106	52/85	4
Prospect Park	Brooklyn	40.6606, −73.9712	0/1	2
Silver Lake Park	Staten Island	40.6276, −74.0932	0/2	2
Van Cortlandt Park	Bronx	40.9020, −73.8823	0/1	2
Willowbrook Park	Staten Island	40.6005, −74.1581	49/72	2
Wolfe’s Pond Park	Staten Island	40.5242, −74.1952	50/60	2

*Sampling effort describes how many visits were made to the park during the sampling period. All ticks were collected from the environment while questing.

### Nymphal Tick Collection

We performed tick collections under a City of New York Parks and Recreation research permit and a New York State Department of Environmental Conservation license to collect. We surveyed parks twice, with a minimum of 2 weeks separation, during the nymphal activity peak (*15*) of May 30–June 30, 2017 (Table 1). All tick collections were conducted by the same 2 persons. We scaled transect coverage by the park area and restricted transects to continuous forest patches that were large enough to complete 100-m transects (Appendix Table 1). Within each park, we divided the effort by 50% along trail/forest edge and 50% perpendicular to the trail into interior forest. We collected ticks every 20 m along the transects by dragging a 1 m^2^ white corduroy cloth (*16*), then removed ticks with forceps, placed them in vials containing 100% ethanol, and identified them to species and life stage by using a standard key (*17*). We recorded global positioning system waypoints at the beginning of each transect and every 20 m. Surveys were not conducted on days with rain.

### Screening of *I. scapularis* Ticks for Infection with *B. burgdorferi*

We screened ≈50 nymphal ticks for *B. burgdorferi* infection in each park (with 1 exception, Bloomingdale Park [n = 39]). We considered this screening conservative because we estimated that >10 ticks should be screened to be 95% confident the site is negative for *B. burgdorferi* if the expected infection prevalence is 26.6%. We homogenized ticks and extracted genomic DNA by using the DNeasy Blood and Tissue Kit (QIAGEN, https://www.qiagen.com) or DNA-zol BD (Molecular Research Center, https://www.mrcgene.com) according to the manufacturers’ recommendations with modifications (*18*). We used PCRs to screen for infection with *B. burgdorferi* by using primer sets for flagellin (*19*), 16S rRNA (*20*), and outer surface protein A (*21*) genes (thermocycling conditions provided in the Appendix). DNA isolated from *B. burgdorferi* strain 2591 culture and from uninfected laboratory-reared ticks were used as positive and negative controls in all PCRs. We identified positive samples by their band size and sequenced amplicon subsamples to confirm the genetic product identity.

### Landscape Analyses

#### Land Cover Layers

We used a high-resolution (1 m × 1 m) land cover dataset for NYC derived from 2010 Light Detection and Ranging (https://catalog.data.gov/dataset?tags=lidar) data and 2008 4-band orthoimagery. Data were classified by using a rule-based expert system into 7 land cover classes: tree canopy, grass/shrub, bare soil, water, buildings, roads, and other paved surfaces (*22*). We combined buildings, roads, and paved surfaces into 1 impervious surface land cover class. We extracted the 24 park polygons from the NYC Open Spaces file (*23*) and quantified the park area and forest area within each park.

#### Land Cover Composition Surrounding Parks

We used buffering, a geographic information system procedure, to extract the proportion of each land cover class within a fixed width area surrounding the park boundaries, excluding coastal waterways. To assess the most predictive buffer size, we calculated the percentage of each land cover class (tree canopy, grass, soil, water, and impervious surfaces) for 5 buffer widths spaced every 100 m from 100 through 500 m. We used the buffer surrounding the park edge as a predictor of *I. scapularis* tick density within the park to indicate the accessibility of the park to hosts carrying feeding ticks or the pathogen.

#### Landscape Connectivity Metrics

For parks in all boroughs, we calculated the Euclidean distance between each pair of park centroids. We set all values in the distance matrix >4.8 km to 0 (distance threshold; i.e., all parks that were >4.8 km were considered unconnected); all park pairs <4.8 km were set to 1. This threshold is based on the average deer movement on Staten Island of 4.0–4.8 km (*24*) and the assumption that new tick populations are established from female adult ticks dropping off of deer. We used the total number of connections between park pairs according to the distance threshold as a model covariate.

We conducted connectivity analyses based on circuit theory only for Staten Island because this borough had a large number of established tick populations with variable densities among parks. We calculated a metric called flow centrality by using the programs Circuitscape (*25*), Linkage Mapper, and Centrality Mapper to assess the importance of each sampled park in maintaining connectivity across the island for deer, ticks, and pathogens (Appendix). We considered the outline of the parks as nodes, or the population sources, and the remaining raster pixels as the matrix. We assigned resistance values to each land cover class in the matrix according to its resistance to deer movement (*26*–*28*) and gene flow (*29*). Although focusing on deer, the resistance values broadly represented connectivity for other known host species of *I. scapularis* ticks and *B. burgdorferi* (Appendix Table 2). We applied Linkage Mapper (*30*), which uses parameters from Circuitscape to identify the least cost paths (LCPs), the single best path of lowest resistance that an animal may use to move through the matrix. We used the LCP network in Centrality Mapper, which assigns each link between nodes a resistance equivalent to the cost-weighted distance of the corresponding LCP. Centrality Mapper applies 1 amp of current into a pair of nodes, iterating through each possible pair of nodes, to calculate the flow centrality score or the current sum across all nodes and connections. Flow centrality is a measure of the contribution of a park to maintaining network connectivity on Staten Island and was used as a covariate in tick density and infection prevalence models.

### Model Development

#### Covariate Standardization and Buffer Size Selection 

We developed 1 model to examine the presence of *I. scapularis* ticks in parks across all NYC boroughs and 2 models with only Staten Island data to determine the best predictors of *I. scapularis* tick density and nymphal infection prevalence. We standardized all landscape covariates used in the 3 models by subtracting the mean and dividing by 1 SD. To determine the most predictive buffer size of *I. scapularis* nymphs, we ran univariate negative binomial generalized linear models (GLMs; glm.nb in the MASS package [*31*] in R [*32*]) for all land cover buffer sizes. We included an offset term, the natural log of the total transect length in a given park, to account for sampling effort. We compared the univariate models of the 5 buffer sizes for each land cover class by using the Akaike Information Criterion (AIC) scores (*33*) and retained the buffer size with the lowest AIC for future analyses (Appendix Table 3).

**Table 3:** *Ixodes scapularis* tick nymphal infection prevalence for *Borrelia burgdorferi* in study of enhancement of Lyme disease risk by landscape connectivity, New York, New York, USA*

Park	No. nymphs positive/no. tested	Site NIP
Bloomingdale Park	5/39	0.128
Blue Heron Park	22/54	0.407
Clay Pit Ponds State Park Preserve	11/52	0.211
Conference House Park	12/51	0.235
Freshkills Park	12/57	0.210
High Rock Park	13/51	0.254
Latourette Park	30/105	0.285
Pelham Bay Park	21/52	0.403
Willowbrook Park	4/49	0.081
Wolfe’s Pond Park	19/50	0.380
Total	149/560	0.266

*Screening results for *B. burgdorferi* infection in nymphal *I. scapularis* ticks from 1 park in the Bronx and 9 parks on Staten Island. Ticks were screened from parks with >39 collected ticks. NIP, nymphal infection prevalence.

### Model Selection

We used GLMs (binomial and negative binomial families) without interactions or random effects to examine the NYC-wide and Staten Island data. For all global models, we assessed multicolinearity between the covariates by using the variance inflation factor (VIF) (*34*) and retained covariates for each final analysis that had a VIF score <4 (*34*). We used an information theoretic approach (*33*) and AIC for small sample sizes (AICc) to identify the best-fitting models describing presence, density, and infection prevalence of *I. scapularis* ticks. We used multimodel inference, which uses model averaging (MuMIn package [*35*] in R [*32*]) to include information from competing models that significantly explain the data. The averaged model is based on a subset of models within 95% of the cumulative AIC weights. The relative importance (RI) of each covariate ranges from 0 through 1 and describes the sum of the Akaike weights in each model in which the covariate is present. If there were no closely competing models (within ∆AIC <2 from the lowest AICc score), we did not use model averaging and selected the final candidate model with the lowest AICc score. We evaluated model fit with McFadden R^2^ (*36*) for logistic regression models and assessed the root mean squared error (RMSE) for the negative binomial model. We included the same offset term as above in all models.

### *I. scapularis* Nymphs in NYC

We used a binomial GLM to examine drivers of presence of *I. scapularis* ticks at parks throughout the 5 boroughs. We considered established parks those where >6 ticks were collected during 2 surveys. This threshold was used by Dennis et al. (*37*) and Eisen et al. (*38*) to classify US counties and was meant to distinguish reproductive tick populations from individual immature ticks that might have detached from a bird. Covariates used to model tick presence were tree canopy area within the park (square meters); the number of connections to other parks within 4.8 km (range 0–5 connections); the land cover composition of the park buffers, including tree canopy, impervious surfaces, water, grass/shrub (percentage within 100 m of park edge); and soil (percentage within 300 m of park edge).

### Density of *I. scapularis* Nymphs on Staten Island, NY

**Figure 2:**
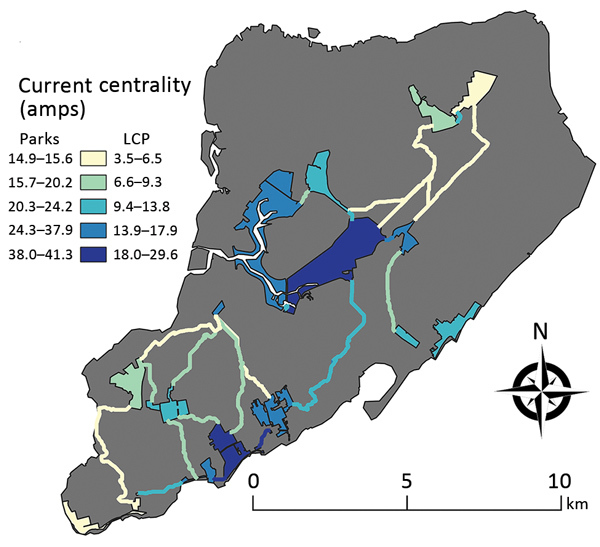
Current centrality for parks and linkages on Staten Island, New York, USA, 2017. In connectivity analysis, the park outlines were used as nodes, and gray indicates the matrix used for the resistance layer. The parks and linkages are color-graded according to their centrality values. Lighter colors indicate lower centrality, and darker colors indicate higher centrality for the network. Amps is the unit used to describe the flow of charge through the nodes. LCP, least cost path.

We used a negative binomial GLM to examine relationships between landscape metrics and tick density (nymphal count/transect length). The negative binomial error structure was selected by using a likelihood ratio test that compared the fit with a poisson error structure. The covariates included in the models were the same as the presence/absence model for NYC, with the addition of the flow centrality scores (range 14.9–41.3) (Figure 2). We examined the global spatial autocorrelation of residuals from the tick density regression model by using a Moran I test (*39*).

### Prevalence of *I. scapularis* Nymphs Infected with *B. burgdorferi* on Staten Island

We used a binomial GLM model to assess the best covariates to predict the nymphal infection prevalence (NIP) at 9 parks on Staten Island. The covariates included in the models were the same as those used in the nymphal density model for Staten Island in addition to the density of nymphs.

## Results

### Buffer Size Selection

The coefficient signs stayed constant for all buffer sizes within the same land cover class in the univariate GLMs (Appendix Table 3). All models examining percentage of tree canopy in the buffer showed a positive effect on tick density; all models that included percentage of grass, soil, water, and impervious surfaces in the buffer had negative effects on tick density. A buffer size of 100 m was the best fit for all land cover classes except bare soil, for which a 300 m buffer had the lowest AIC (Appendix Table 3).

### *I. scapularis* Ticks in NYC

At least 1 *I. scapularis* nymph was found at 17 of 24 parks surveyed throughout NYC. Of these parks, 10 had >6 nymphs and were categorized as established for *I. scapularis* populations; all of these sites were on Staten Island, except for Pelham Bay Park in the Bronx (Table 1). The model with the lowest AIC included the number of connections a park had to surrounding parks within 4.8 km (p = 0.005) (Appendix Figure 1). This model explained moderate levels of variation with a McFadden R^2^ of 0.38.

### Density of *I. scapularis* Ticks on Staten Island

Because 9 of 10 parks with established tick populations were on Staten Island, we limited the analysis of *I. scapularis* density to this borough. Blacklegged ticks were most abundant in the central and southern regions of the island (Figure 1). We removed 1 covariate that had a VIF >4, percentage of grass in the buffer. We identified 4 multivariate models with considerable support (∆AIC <2), these were within 95% of the cumulative AIC weights that composed the averaged model. Flow centrality, percentage tree canopy, soil, and water within the buffer were the major covariates (RI = 1.00) and were present in all 4 models comprising the averaged model (Table 2; Appendix Table 4). The percentagew impervious surfaces within a 100-m buffer (RI = 0.36) and the tree canopy area within the park (RI = 0.65) showed no major effect on *I. scapularis* tick density (CIs include 0) in the averaged model, although tree canopy area showed major positive effects in a subset of models that comprised the averaged model. The RMSE of the model residuals was 1.04; a total of 64.8% of the values fell within 1 RMSE and 98.9% of the values fell within 2 RMSE. Residuals from the regression model were not spatially autocorrelated according to the Moran I test results (p = 0.09), indicating the tick surveys can be considered independent.

**Table 2:** Averaged model for *Ixodes scapularis* tick density in study of enhancement of Lyme disease risk by landscape connectivity, New York, New York, USA*

Variable	Coefficient estimate	95% CI	RI
Intercept	−3.0262	−3.20 to −2.84	NC
Flow centrality, amps	0.4058	0.13 to 0.67	1.00
Tree canopy area in park, m^2^	0.1821	−0.001 to 0.55	0.65
% Trees†	0.5068	0.27 to 0.73	1.00
% Impervious†‡	0.0454	−0.13 to 0.38	0.36
% Water†	−0.4285	−0.64 to −0.20	1.00
% Soil§	−0.5684	−0.88 to −0.25	1.00

*Values are for 13 parks on Staten Island. If the CI includes 0, there was no significant effect of the covariate on tick density. NC, not considered; RI, relative importance. †Within 100-m buffer. ‡Buildings, roads, and paved surfaces. §Within 300-m buffer.

### Prevalence of *I. scapularis* Nymphs Infected with *B. burgdorferi* on Staten Island

We estimated NIP for 9 parks on Staten Island and 1 park in the Bronx (Table 3). A total of 8%–40% of ticks tested at each site were positive for *B. burgdorferi* (Table 3; Figure 1), and the average NIP across all sites was 26.6% (149/560) positive for *B. burgdorferi*.

**Figure 3:**
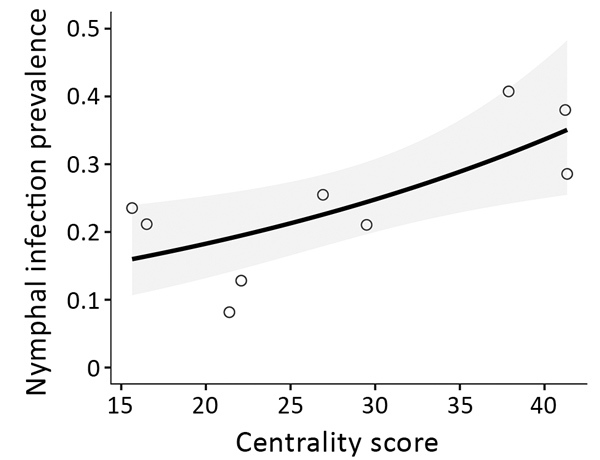
*Ixodes scapularis* tick nymphal infection prevalence and flow centrality model for Staten Island, New York, USA, 2017. The centrality score of 9 parks was the best predictor for nymphal infection prevalence. Shown are results of the binomial generalized linear model (p = 0.009). SE (± 0.1040) is indicated in gray. The coefficient estimate is 0.2714.

We assessed the VIF of the global model limited to the NIP for Staten Island and removed 2 covariates with VIF scores >4, percentage of tree canopy within a 100-m buffer, and density of nymphs. The univariate model with flow centrality had a model weight of 0.45, and no other model combinations were within <2 ∆AIC from the lowest AIC. Therefore, we did not apply model averaging and determined that flow centrality was the significant factor (p = 0.009) in predicting NIP at parks on Staten Island (Figure 3). The McFadden R^2^ for this model showed low explained variation (R^2^ = 0.13), likely caused by small sample size.

## Discussion

We examined how urban landscape composition and configuration reflects the environmental and ecologic conditions driving the distribution of ticks and their pathogens. We found the distance between urban parks best explained whether *I. scapularis* ticks were present and that flow centrality of parks, an indicator of the connectivity of parks for deer and other hosts, had the largest positive effect on the density and infection prevalence of *I. scapularis* nymphs. Covariates that describe the composition of the landscape surrounding each park also had a major positive (percentage tree canopy) or negative (percentage water and soil) effect on the densities of *I. scapularis* nymphs.

Environmental conditions can limit *I. scapularis* tick population establishment and persistence. The negative effect of bare soil surrounding parks might reflect deer aversion to open habitats and tick physiology. To prevent desiccation, ticks seek microclimates that have higher ambient humidity (*40*). Ticks achieve water replenishment when they descend to lower vegetation layers (*40*), often leaf litter. Without vegetation, ticks might quickly desiccate when dropped in bare soil. Furthermore, the amount of water surrounding the park negatively affected *I. scapularis* tick density, suggesting that water might also serve as a barrier for deer movement into particular parks and could limit tick population persistence. Our findings are consistent with studies that have found agricultural fields, large urbanized areas (*27*), and a combination of landscape features with low permeability, such as waterways and roads, to impede deer movement (*29*), potentially slowing tick expansion.

The introduction of *I. scapularis* ticks into new habitats can occur through multiple pathways. Because these ticks move only a few meters during each life stage, host movement and habitat use during tick feeding determine dispersal patterns (*41*). Landscape structure and connectivity might differentially affect reservoir (rodents, medium-size mammals, and birds) and reproductive host (deer) movement. Adult *I. scapularis* ticks are mainly distributed by deer (*41*); they serve as the primary host for adult ticks, and >90% of female *I. scapularis* ticks feed on deer (*42*). Although locally dispersing or migrating passerine birds play a role in moving immature ticks longer distances (*43*), deer are key hosts for establishing new populations locally because 1 female adult tick will lay ≈2,000 eggs after a successful blood meal from a deer (*44*). We were unable to sample larvae and adults; however, a previous study has shown a positive linear relationship between the density of larvae and nymphs (*9*). Without sufficient deer available, tick populations cannot be sustained or are sustained at much lower levels (*42*). The lack of deer reported in parks where we did not find established *I. scapularis* tick populations (*45*) indicates a strong link between deer and presence of *I. scapularis* ticks in NYC parks.

Landscape structure and connectivity also impacts the distribution and movement of small and medium-size mammals that are hosts for immature ticks and *B. burgdorferi*. Rodent hosts can contribute to a slower range expansion of *B. burgdorferi* and infected immature ticks (*46*). Rodent movement is determined by their ability to penetrate habitats, and sources of landscape resistance imposed on movement of white-footed mice, Eastern chipmunks, and red squirrel is similar between these host species (*47*).

Our results show that landscape composition and configuration have direct implications on urban Lyme disease risk. This finding is especially useful because >80% of persons in North America now reside in urban centers (*48*), the distribution of *I. scapularis* ticks continues to expand (*38*), and interest is increasing in urban green space serving as a key moderator of poverty, health, health equity, and environmental justice (*49*). Initiatives that increase urban green space have clear benefits for human well-being, climate change mitigation, and wildlife conservation. However, our study calls attention to the need to understand the drivers of tick distribution and densities within urban green spaces in the United States. Our findings on the role of flow centrality in maintaining tick and pathogen populations indicate a potential nonlinear effect of forest fragmentation on tickborne disease risk by emphasizing that fragment connectivity is a neglected key factor (however, see reports by Mechai et al. [*47*] and McClure and Diuk-Wasser [*50*]). A better understanding of how landscape shapes host communities, their movement, and tick habitat in urban and suburban regions is critical to ameliorate the risk for tickborne diseases.

Appendix Additional information on enhancement of entomologic risk for Lyme disease by landscape connectivity, New York, New York, USA.
