# Supplementary material for: Enhancement of Risk for Lyme Disease by Landscape Connectivity, New York, New York, USA
Source: Emerg Infect Dis. 2019 Jun;25(6):1136–43. doi: 10.3201/eid2506.181741 (PMC6537717; doi:10.3201/eid2506.181741)
Supplement: Appendix — Additional information on enhancement of entomologic risk for Lyme disease by landscape connectivity, New York, New York, USA. [file 18-1741-Techapp-s1.pdf]

Article DOI: https://doi.org/10.3201/eid2504.181741

# Enhancement of Risk for Lyme Disease by Landscape Connectivity, New York, New York, USA

## Appendix

### Screening of *Ixodes scapularis* Ticks for Infection with *Borrelia burgdorferi*

Screening of nymphal *Ixodes scapularis* ticks was conducted by extracting genomic DNA and subsequent PCR by using the DNeasy Blood and Tissue Kit (QIAGEN, https://www.qiagen.com) or DNA-zol BD (Molecular Research Center, https://www.mrcgene.com) according to the manufacturers' recommendations with modifications (*1*). In brief, ticks were washed twice with autoclaved deionized water and homogenized in microtubes containing 400 µL DNA-zol BD by using the extended end of an autoclaved large paper clip or a copper BB and a vibration mill (Model MM301; Retsch, https://www.retsch.com). The homogenates were centrifuged at 14,000 × *g* for 10 min, and supernatant was transferred into a new microtube. After adding 3 µL of Poly Acryl Carrier (Molecular Research Center) to the supernatant, DNA was then precipitated by using absolute ethanol. The DNA pellet was washed twice with 75% ethanol, air-dried briefly, reconstituted in 30 µL of 1 × TE buffer (10 mmol/L Tris-HCl [pH 8.0], 1 mmol/L EDTA), and stored at –20°C for further analysis.

Isolated DNA from the ticks served as templates in subsequent PCRs to screen for infection with *B. burgdorferi* by using specific primer sets for flagellin (*2*), the 16S rRNA (*3*), and outer surface protein A (*4*) genes. DNA isolated from *B. burgdorferi* cultures was used as a positive control, and DNA from uninfected laboratory-reared ticks was used as a negative control in all PCRs. The positive control used was DNA isolated from a *B. burgdorferi* strain 2591 (*5*) culture. A Taq PCR Core Kit (QIAGEN) was used for all PCRs according to the manufacturer's recommendation. A 50-µL reaction volume was prepared with 3 µL template DNA, 4 µL each primer (0.1–0.5 µmol/L), 5 µL 10× QIAGEN PCR Buffer (containing 15 mmol/L $MgCl_2$), 1 µL

dNTP mixture (10 mmol/L of each dNTP), 0.25 μL Taq DNA polymerase (1.25 U/reaction), and 32.75 μL water.

PCR cycling conditions for flagellin were an initial reaction activation step of 95°C for 3 min, followed by 40 cycles of denaturation at 95°C for 1 min, annealing at 55°C for 1 min, and extension at 72°C for 1 min. The final cycle was a 5-min extension at 72°C. Cycling conditions for 16S rRNA were an initial reaction activation step of 95°C for 10 min, followed by 35 cycles of denaturation at 95°C for 1 min, annealing at 54°C for 1 min, and extension at 72°C for 1 min and 20s. The final cycle was completed with extension for 7 min at 72°C. Cycling conditions for the outer surface protein A gene were a reaction activation step at 94°C for 10 min, followed by 45 cycles of denaturation at 95°C for 1 min, annealing at 50°C for 45s, and extension at 72°C for 2 min. The final cycle was completed with extension for 7 min at 72°C.

All PCRs were performed with Veriti or the GeneAmp PCR System 9700 (Applied Biosystems, https://www.thermofisher.com/us/en/home/brands/applied-biosystems.html). PCR-amplified products were subjected to electrophoresis on 0.6%–1.2% agarose gels, stained with ethidium bromide, visualized under UV light, and documented by using the GelDoc System (UVP, https://www.uvp.com). A randomly selected subsample of the PCR products was selected from the positive control and the sample for sequencing to ensure the product corresponded to the correct genes. When $\geq$2 of the 3 genetic markers for *B. burgdorferi* were identified in the sample, the sample was identified as being positive.

### Landscape Connectivity Metrics

Circuitscape (https://circuitscape.org) simulates electrical currents through circuit networks that represent potential animal movement across habitat types with varying levels of resistance. The 2 inputs into the Circuitscape program are a shapefile that defines the nodes, or the sources of the populations, and a raster file of the landscape where land cover types and environmental attributes have different levels of resistance for animal movement. We included the 13 parks we sampled on Staten Island, NY, USA, as nodes. We parameterized the raster layer for input into Circuitscape according to the resistance values each land cover class in the matrix imposes on white-tailed deer movement. The assigned resistance values were based on studies of deer movement (*6*–*8*) and deer gene flow (*9*–*11*) (Appendix Table 2). We applied Linkage Mapper (*12*), which uses parameters from Circuitscape, to identify the least cost paths between

neighboring core areas. The least cost paths network was then read into the Centrality Mapper (*13*) program to assign each link between nodes a resistance that equals the cost-weighted distance of the corresponding least cost path (*13*). Centrality Mapper then applies 1 amp of current into a pair of nodes, iterating through each possible pair of nodes, to calculate the sum of the current across all nodes and connections. From the Centrality Mapper analysis, we obtained a flow centrality score for each sampled park on Staten Island that was used as a covariate in the tick abundance and infection prevalence models.

### Circuitscape Analysis for White-Footed Mice (*Peromyscus leucopus*)

We ran Circuitscape and used white-footed mice resistance values (Appendix Table 5) to examine the relationship between flow centrality according to deer and mice resistance on nymphal infection prevalence. There was no major relationship between mice centrality and the nymphal infection prevalence although the major relationship between deer centrality and nymphal infection prevalence remained (Appendix Table 6). This finding might be caused by the fact that the resistance values used for deer represent those that would be applied to other reservoir hosts for *Borrelia burgdorferi* that require similar habitats to that of deer.

### References


<jrn>1. Molaei G, Andreadis TG, Armstrong PM, Anderson JF, Vossbrinck CR. Host feeding patterns of *Culex* mosquitoes and West Nile virus transmission, northeastern United States. Emerg Infect Dis. 2006;12:468–74. PubMed http://dx.doi.org/10.3201/eid1203.051004</jrn>

<jrn>2. Barbour AG, Maupin GO, Teltow GJ, Carter CJ, Piesman J. Identification of an uncultivable *Borrelia* species in the hard tick *Amblyomma americanum*: possible agent of a Lyme disease-like illness. J Infect Dis. 1996;173:403–9. PubMed http://dx.doi.org/10.1093/infdis/173.2.403</jrn>

<jrn>3. Gazumyan A, Schwartz JJ, Liveris D, Schwartz I. Sequence analysis of the ribosomal RNA operon of the Lyme disease spirochete, *Borrelia burgdorferi*. Gene. 1994;146:57–65. PubMed http://dx.doi.org/10.1016/0378-1119(94)90833-8</jrn>

<jrn>4. Persing DH, Telford SR III, Spielman A, Barthold SW. Detection of *Borrelia burgdorferi* infection in *Ixodes dammini* ticks with the polymerase chain reaction. J Clin Microbiol. 1990;28:566–72. PubMed</jrn>

<jrn>5. Anderson JF, Magnarelli LA, Burgdorfer W, Barbour AG. Spirochetes in *Ixodes dammini* and mammals from Connecticut. Am J Trop Med Hyg. 1983;32:818–24. PubMed http://dx.doi.org/10.4269/ajtmh.1983.32.818</jrn>

<jrn>6. Girardet X, Conruyt-Rogeon G, Foltête J-C. Does regional landscape connectivity influence the location of roe deer roadkill hotspots? Eur J Wildl Res. 2015;61:731–42. http://dx.doi.org/10.1007/s10344-015-0950-4</jrn>

<jrn>7. Kelly AC, Mateus-Pinilla NE, Brown W, Ruiz MO, Douglas MR, Douglas ME, et al. Genetic assessment of environmental features that influence deer dispersal: implications for prion-infected populations. Popul Ecol. 2014;56:327–40. http://dx.doi.org/10.1007/s10144-013-0427-9</jrn>

<jrn>8. Long ES, Difenbach DR, Rosenberry CS, Wallingford BD, Grund MD. Forest cover influences dispersal distance of white-tailed deer. J Mammal. 2005;86:623–9. http://dx.doi.org/10.1644/1545-1542(2005)86[623:FCIDDO]2.0.CO;2</jrn>

<jrn>9. Robinson SJ, Samuel MD, Lopez DL, Shelton P. The walk is never random: subtle landscape effects shape gene flow in a continuous white-tailed deer population in the midwestern United States. Mol Ecol. 2012;21:4190–205. PubMed http://dx.doi.org/10.1111/j.1365-294X.2012.05681.x</jrn>

<jrn>10. Lang KR, Blanchong JA. Population genetic structure of white-tailed deer: understanding risk of chronic wasting disease spread. J Wildl Manage. 2012;76:832–40. http://dx.doi.org/10.1002/jwmg.292</jrn>

<jrn>11. Blanchong JA, Sorin AB, Scribner KT. Genetic diversity and population structure in urban white-tailed deer. J Wildl Manage. 2013;77:855–62. http://dx.doi.org/10.1002/jwmg.521</jrn>

<bok>12. McRae B, Kavanagh D. Linkage mapper connectivity analysis software. Fort Collins (CO): The Nature Conservancy; 2011 [cited 2019 Jan 17]. https://circuitscape.org/linkagemapper/</bok>

<bok>13. McRae B. Centrality mapper connectivity analysis software. Fort Collins (CO): The Nature Conservancy; 2012</bok>

<jrn>14. Rizkalla CE, Swihart RK. Incorporating behavior-based indices of connectivity into spatially explicit population models. Can J Zool. 2012;90:222–36. http://dx.doi.org/10.1139/z11-130</jrn>

<jrn>15. Kierepka EM, Anderson SJ, Swihart RK, Rhodes OE Jr. Evaluating the influence of life-history characteristics on genetic structure: a comparison of small mammals inhabiting complex agricultural landscapes. Ecol Evol. 2016;6:6376–96. PubMed http://dx.doi.org/10.1002/ece3.2269</jrn>

<jrn>16. Marrotte RR, Gonzalez A, Millien V. Landscape resistance and habitat combine to provide an optimal model of genetic structure and connectivity at the range margin of a small mammal. Mol Ecol. 2014;23:3983–98. PubMed http://dx.doi.org/10.1111/mec.12847</jrn>

**Appendix Table 1.** Park areas and transect lengths sampled for *Ixodes scapularis* ticks, New York, NY, USA

| Park area, ha | Transect coverage, $m^2$ |
|---|---|
| 25–78 | 550 |
| 79–111 | 700 |
| 112–257 | 850 |
| 258–380 | 1,000 |

**Appendix Table 2.** Circuitscape resistance values for parks sampled for *Ixodes scapularis* ticks, New York, NY, USA*

| Land cover class | Resistance value |
|---|---|
| Tree canopy | 1 |
| Grassland/shrub | 1 |
| Bare soil | 30 |
| Water | 100 |
| Paved surfaces | 100 |
| Roads/railroads | 300 |
| Buildings | 1,000 |

*Resistance values for deer were assigned to each land cover class used in Circuitscape. Resistance values were derived from Girardet et al. (*6*).

**Appendix Table 3.** Buffer spatial scales used to model the abundance of *Ixodes scapularis* nymphs, New York, NY, USA*

| Land cover class | Coefficient estimate | p value | Buffer size, m | ΔAIC |
|---|---|---|---|---|
| Tree canopy | | | | |
| | **0.80** | **<0.001** | **100** | – |
| | 0.65 | <0.001 | 200 | 11.77 |
| | 0.48 | <0.001 | 300 | 19.82 |
| | 0.38 | <0.001 | 400 | 23.44 |
| | 0.31 | 0.005 | 500 | 25.36 |
| Grassland/shrub | | | | |
| | **–0.27** | **0.013** | **100** | – |
| | –0.14 | 0.189 | 200 | 2.62 |
| | –0.08 | 0.476 | 300 | 3.43 |
| | –0.07 | 0.532 | 500 | 3.46 |
| | –0.06 | 0.572 | 400 | 3.55 |
| Bare soil | | | | |
| | **–1.52** | **<0.001** | **300** | – |
| | –0.95 | <0.001 | 200 | 15.17 |
| | –0.79 | <0.001 | 100 | 17.28 |
| | –0.87 | <0.001 | 400 | 17.97 |
| | –0.62 | <0.001 | 500 | 30.45 |
| Water | | | | |
| | **–0.55** | **<0.001** | **100** | – |
| | –0.46 | <0.001 | 200 | 3.35 |
| | –0.63 | <0.001 | 500 | 4.57 |
| | –0.52 | <0.001 | 400 | 5.35 |
| | –0.41 | <0.001 | 300 | 7.56 |
| Impervious surfaces | | | | |
| | **–0.28** | **0.009** | **100** | – |
| | –0.22 | 0.046 | 200 | 2.56 |
| | –0.15 | 0.165 | 300 | 4.66 |
| | –0.11 | 0.325 | 400 | 5.83 |
| | –0.05 | 0.607 | 500 | 6.70 |

*Shown are results from the univariate negative binomial generalized linear models used to assess the best spatial scale for the land cover buffers to describe tick abundance. The buffer sizes were assessed through AIC comparison of all 5 buffer sizes for each land cover class. The buffer scale that resulted in the lowest AIC is in bold. The ΔAIC column shows the difference in AIC values between the model with the lowest AIC (indicated by –) and all other nested models within 2 AIC. AIC, Akaike's Information Criterion.

**Appendix Table 4.** Summary of the 4 best-fitting models for *Ixodes scapularis* tick abundance, New York, NY, USA*

| Model ID | Variable | Coefficient estimate | SE | p value | AIC | Weight |
|---|---|---|---|---|---|---|
| 124 | Intercept | –3.026 | 0.09068 | <0.001 | 1,092.8 | 0.38 |
| | % soil, 300 m | –0.548 | 0.13900 | <0.001 | | |
| | Centrality | 0.373 | 0.12974 | 0.003 | | |
| | Tree canopy area in park | 0.248 | 0.13349 | 0.06 | | |
| | % water, 100 m | –0.409 | 0.10618 | <0.001 | | |
| | % tree canopy, 100 m | –0.466 | 0.09483 | <0.001 | | |
| 128 | Intercept | –3.031 | 0.08984 | <0.001 | 1,093.5 | 0.27 |
| | % soil, 300 m | –0.470 | 0.14588 | 0.001 | | |
| | Centrality | 0.332 | 0.13108 | 0.01 | | |
| | Tree canopy area in park | 0.320 | 0.14294 | 0.02 | | |
| | % water, 100 m | –0.384 | 0.10673 | <0.001 | | |
| | % tree canopy, 100 m | 0.561 | 0.13576 | <0.001 | | |
| | % impervious, 100 m | 0.153 | 0.12415 | 0.217 | | |
| 122 | Intercept | –3.023 | 0.09220 | <0.001 | 1,093.6 | 0.25 |
| | % soil, 300 m | –0.669 | 0.13343 | <0.001 | | |
| | Centrality | 0.498 | 0.08924 | <0.001 | | |
| | % water, 100 m | –0.484 | 0.09741 | <0.001 | | |
| | % tree canopy, 100 m | 0.502 | 0.09588 | <0.001 | | |
| 126 | Intercept | –3.023 | 0.09214 | <0.001 | 1,095.6 | 0.09 |
| | % soil, 300 m | –0.662 | 0.13857 | <0.001 | | |
| | Centrality | 0.499 | 0.09018 | <0.001 | | |
| | % water, 100 m | –0.483 | 0.09737 | <0.001 | | |
| | % tree canopy, 100 m | 0.526 | 0.13487 | <0.001 | | |
| | % impervious, 100 m | 0.041 | 0.11724 | 0.724 | | |

*Shown are best selected models for *I. scapularis* tick abundance response variable (based on lowest AIC values and cumulative AIC weight). Modeling was performed by using generalized linear models with standardized explanatory variables. All covariates had variable inflation factor multicollinearity scores <4. Descriptive statistics include coefficient estimates, p values represent significance of each predictor variable in the model, the model's AIC score, and model weight. All models make up a cumulative AIC weight >0.95 and were used in the averaged model. The total AIC weight was 0.99. AIC, Akaike's Information Criterion; ID, identification.

**Appendix Table 5.** Circuitscape resistance values for white-footed mice (*Peromyscus leucopus*)*

| Land cover class | Resistance value |
|---|---|
| Tree canopy | 1 |
| Grassland/shrub | 300 |
| Bare soil | 600 |
| Water | 1,000 |
| Paved surfaces | 900 |
| Roads/railroads | 800 |
| Buildings | 900 |

*Resistance values were assigned to each land cover class used in Circuitscape (https://circuitscape.org). Resistance values were derived from multiple sources (*14–16*).

**Appendix Table 6.** Model results for nymphal infection prevalence on Staten Island, New York, USA*

| Predictor | Coefficient estimate | SE | p value |
|---|---|---|---|
| Mice centrality | –0.1929 | 0.1432 | 0.1779 |
| Deer centrality | 0.4156 | 0.1484 | 0.0051 |

*Shown are generalized linear model results for deer and mice centrality as predictors of nymphal infection prevalence. Deer centrality remained significant and mice centrality was not found to be a significant predictor.

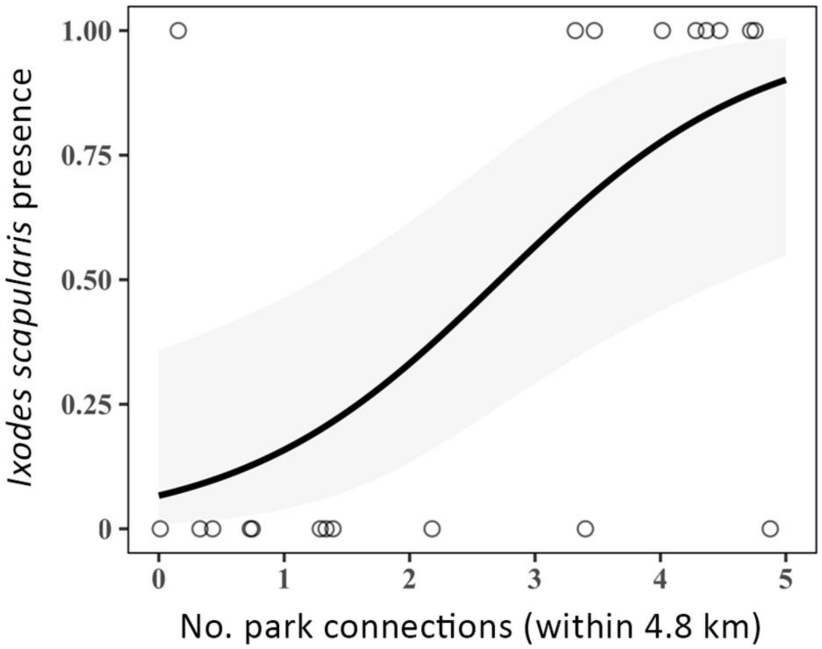


**Appendix Figure.** Number of park connections within 4.8 km of each park showing a strong correlation with presence of *Ixodes scapularis* ticks at sampled sites, New York, NY, USA. Shown are results of the binomial generalized linear model (p = 0.005). SE (± 0.6787) is indicated in gray. The coefficient estimate is 1.8912.
